# Nampt promotes osteogenic differentiation and lipopolysaccharide-induced interleukin-6 secretion in osteoblastic MC3T3-E1 cells

**DOI:** 10.18632/aging.202434

**Published:** 2021-02-01

**Authors:** Shan He, Hanxiang Zhang, Yang Lu, Zhaosi Zhang, Xiang Zhang, Nian Zhou, Zhenming Hu

**Affiliations:** 1 Department of Orthopaedic Surgery, The First Affiliated Hospital of Chongqing Medical University, Chongqing 400016, China; 2 School of Nursing, Chongqing Medical University, Chongqing 400016, China; 3 Department of Orthopaedic Surgery, Affiliated Hospital of Zunyi Medical University, Zunyi 563000, Guizhou, China; 4 Department of Cerebrovascular Diseases, the First Affiliated Hospital of Zunyi Medical University, Zunyi 563000, Guizhou, China; 5 Department of Orthopaedics, Hunan Province People’s Hospital, Changsha 410005, China

**Keywords:** Nampt, Sirt1, IL-6, NF-κB, inflammation

## Abstract

The Nicotinamide phosphoribosyltransferase (Nampt)-NAD-Sirt1 pathway modulates processes involved in the pathogenesis of multiple diseases by influencing inflammation. This study aimed to explore the effect of Nampt in osteogenic differentiation and inflammatory response of osteoblastic MC3T3-E1 cells. We developed an *in vitro* model of lipopolysaccharide (LPS)-induced inflammation and showed that Nampt and Sirt1 were significantly upregulated in LPS-treated MC3T3-E1 cells. LPS induced secretion of the proinflammatory cytokine interleukin-6 (IL-6) and attenuated osteogenic differentiation. Then we transfected cells with adenoviruses to knock down or over express Nampt. Nampt promoted the expression of IL-6, TAK1 and phospho-NF-κB p65 after LPS treatment. Overexpression of Nampt overrode the effect of LPS and rescued LPS-induced inhibition on osteogenic differentiation. FK866, a Nampt inhibitor, had the same inhibitory effect as Nampt knockdown. In addition, Sirt1 suppression by EX527 decreased IL-6 secretion and NF-κB activation without changing the level of Nampt. EX527 also decreased osteogenic differentiation. Incubation with NMN or SRT 1720 also counteract the inhibitory effect of LPS and rescued osteoblast differentiation. Therefore, we demonstrated that Nampt acted both in promoting osteoblast differentiation and in enhancing inflammatory response, mediated by Sirt1 in MC3T3-E1 cells.

## INTRODUCTION

The development and maintenance of the skeletal system is a dynamic process that is regulated by bone remodeling and modeling [1]. Proper balance is achieved by the synchronized activities of multiple cellular participants [2]. Many chronic inflammatory conditions lead to excessive bone resorption as well as impaired bone formation, resulting in systemic osteoporosis and increased fracture rates [3, 4]. Osteoporotic fractures are common in women after age 55 and men after age 65, resulting in bone-associated morbidities and increased mortality in the population [5].

Ageing is accompanied by a state of low-grade chronic inflammation [6]. Proinflammatory cytokines have profound effects on the differentiation and activity of osteoblasts [7]. These cytokines include interleukin-1 (IL-1), tumor necrosis factor (TNF) and interleukin-6 (IL-6). Biological therapies can effectively reduce inflammation, but often does not eliminate it, and therapeutics achieves a state of persistent remission in only a small percentage of patients [8]. Therefore, modulating inflammatory signaling is a promising strategy for bone regeneration. IL-6, the most abundant cytokine in the circulation, may have a major role in bone metabolism [9]. Thus, combating IL-6-induced bone loss is an important and clinically relevant question.

Nampt (also known as visfatin and pre-B-cell colony-enhancing factor (PBEF)) is the rate-limiting enzyme that converts nicotinamide to nicotinamide mononucleotide (NMN) in the nicotinamide adenine dinucleotide (NAD) salvage pathway of NAD biosynthesis in mammals [10]. Nampt is over expressed in various cancers and inflammatory disorders, including osteoarthritis [11], obesity [12], acute lung injury [13], and inflammatory bowel disease (IBD) [14]. Nampt therefore represents a mediator of innate immunity. Nampt regulates the activity of its downstream proteins, including the histone deacetylase sirtuin-1 (Sirt1), which utilizes NAD as a cofactor [11]. Sirt1 deacetylates specific transcription factors and enzymes to influence their activities [15]. Friebe et al. found that Nampt and Sirt1 mRNA expression levels were significantly increased in lipopolysaccharide (LPS)-induced granulocytes [12]. Despite these findings, the relationship between Nampt, Sirt1, osteogenic differentiation and inflammation of preosteoblasts is poorly understood. We therefore utilized an *in vitro* model of LPS-induced inflammation to investigate how Nampt and Sirt1 affected osteogenic differentiation and inflammatory response of preosteoblasts.

## RESULTS

### LPS induced IL-6 secretion and upregulated Nampt expression

**Figure 1:**
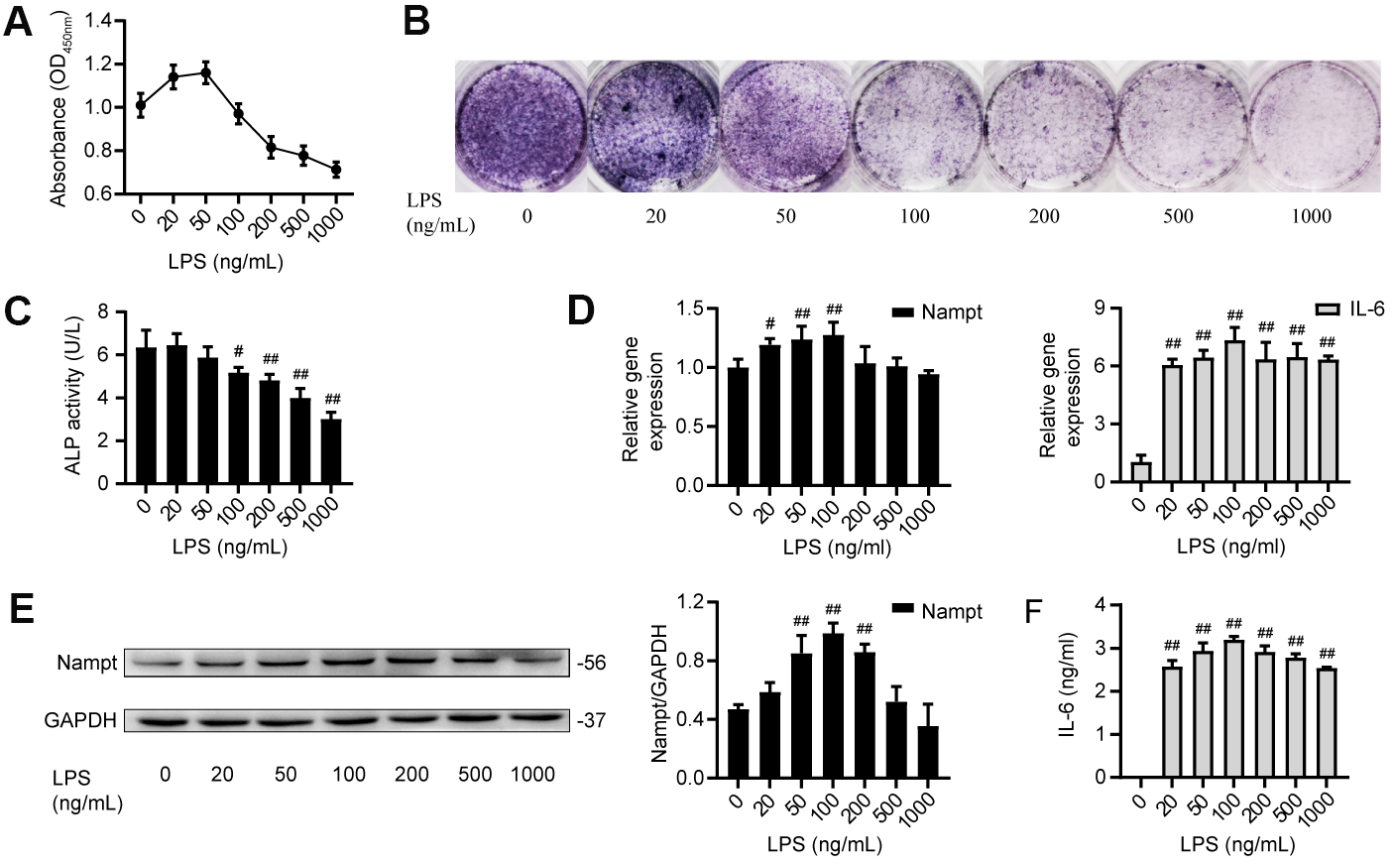
**LPS induced IL-6 secretion and upregulated Nampt expression in MC3T3-E1 cells.** (**A**) CCK-8 results showing that cell proliferation was inhibited when the LPS concentration was above 100 ng/mL. (**B**) ALP staining and (**C**) Measurement of ALP activity showing that ALP was decreased on the 7^th^ day after LPS treatment. On day 3 of LPS treatment, (**D**) Real-time PCR analysis of Nampt and IL-6 mRNA expression normalized to β-actin. (**E**) Western blot results showing the protein level of Nampt. (**F**) IL-6 was induced by LPS at varying concentrations, as measured by ELISA. The data are expressed as the mean ± SD. #, *p*<0.05 vs. LPS 0 ng/mL; ##, *p*<0.01 vs. LPS 0 ng/mL.

MC3T3-E1 cells were cultured with LPS at concentrations of 0, 20, 50, 100, 200, 500 and 1000 ng/mL. Cell counting kit-8 (CCK-8) assay results showed that LPS reduced the proliferation of MC3T3-E1 cells in a dose-dependent manner when at concentrations above 100 ng/mL (Figure 1A). When cultured in osteoblast medium (OBM) with LPS for 7 days, MC3T3-E1 cells showed significant inhibition of osteogenic differentiation in a dose-dependent manner, as indicated by the alkaline phosphatase (ALP) staining and ALP activity (Figure 1B, 1C). After treatment with LPS in OBM for 3 days, the group treated with 100 ng/mL LPS had the highest mRNA expression of Nampt and IL-6 (Figure 1D). Western blot analysis results showed that the group treated with 100 ng/mL LPS had the highest protein expression of Nampt (Figure 1E). Enzyme-linked immunosorbent assay (ELISA) was used to detect proinflammatory cytokines in culture supernatants on day 3. IL-6 was induced, and 100 ng/mL LPS induced the highest IL-6 level in culture supernatants (Figure 1F). Real-time PCR and ELISA did not detect IL-1β and TNF-α after LPS treatment. These results suggested that high concentrations of LPS induced cell death, and 100 ng/mL LPS was an appropriate concentration for subsequent experiments to explore the relationship between Nampt and the inflammatory response.

### Nampt promoted osteogenesis of MC3T3-E1 cells *in vitro*

**Figure 2:**
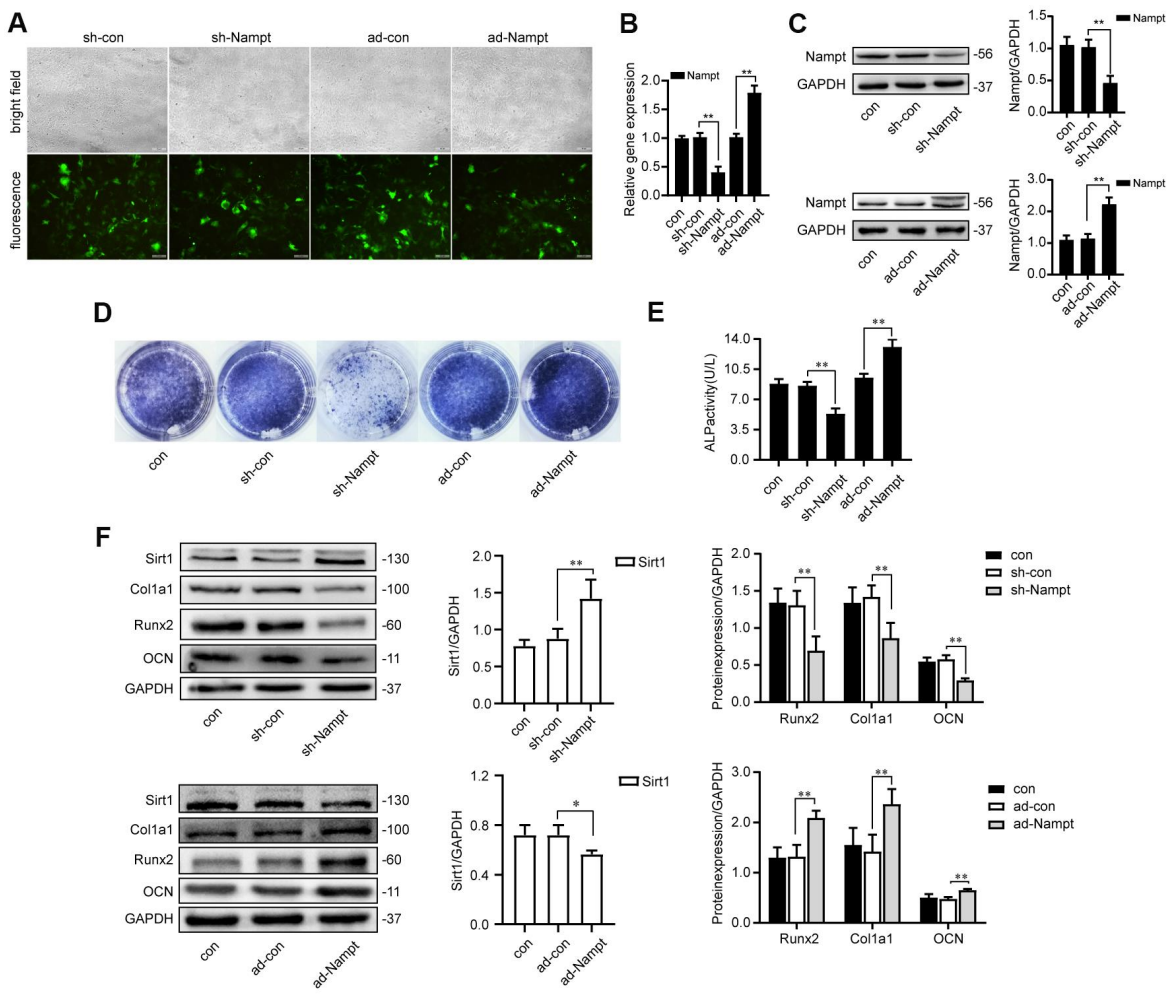
**Nampt promoted osteogenic differentiation.** (**A**) MC3T3-E1 cells were transfected with adenoviruses. GFP marker genes were detected 48 h after transfection under both bright field and fluorescence microscopy (100×). (**B**) Real-time PCR analysis showing Nampt gene expression normalized to β-actin and (**C**) Western blot results showing the protein expression of Nampt 3 days after the transfection. On the 7^th^ d of culture in OBM, (**D**) ALP staining and (**E**) Measurement of ALP activity showing that ALP was decreased in sh-Nampt cells and increased in ad-Nampt cells. (**F**) Western blot results showing the protein expression of Sirt1, Runx2, Col1a1 and OCN. Con (control) cells were not transfected; sh-con and ad-con (negative control) cells were transfected with nontargeting control viruses; sh-Nampt cells were transfected with Nampt knockdown shRNA; ad-Nampt cells were transfected with vector over expressing Nampt. The data are expressed as the mean ± SD. *, *p*<0.05; **, *p*<0.01.

In order to obtain high levels of transgene expression, cells were transfected with sh-Nampt, and ad-Nampt. After 48 h of transfection, cells were observed by fluorescence microscope (Figure 2A). After 3 days, Nampt expression was decreased in sh-Nampt cells and increased in ad-Nampt cells at both the mRNA and protein levels, and this did not occur in cells transfected with nontargeting control viruses (Figures 2B, 2C).

Cells were cultured in medium for 3 days after adenovirus transfection, then cultured in OBM for 7 days. Nampt knockdown decreased the expression of osteogenic markers: ALP, Runx2, alpha 1 type I collagen (Col1a1) and osteocalcin (OCN), while Nampt overexpression increased the expression of these markers, as showed by ALP staining, ALP activity and western blot (Figures 2D–2F). Sirt1 expression was increased after Nampt knockdown, and decreased after Nampt overexpression (Figure 2F).

### Nampt promoted LPS-induced IL-6 secretion

**Figure 3:**
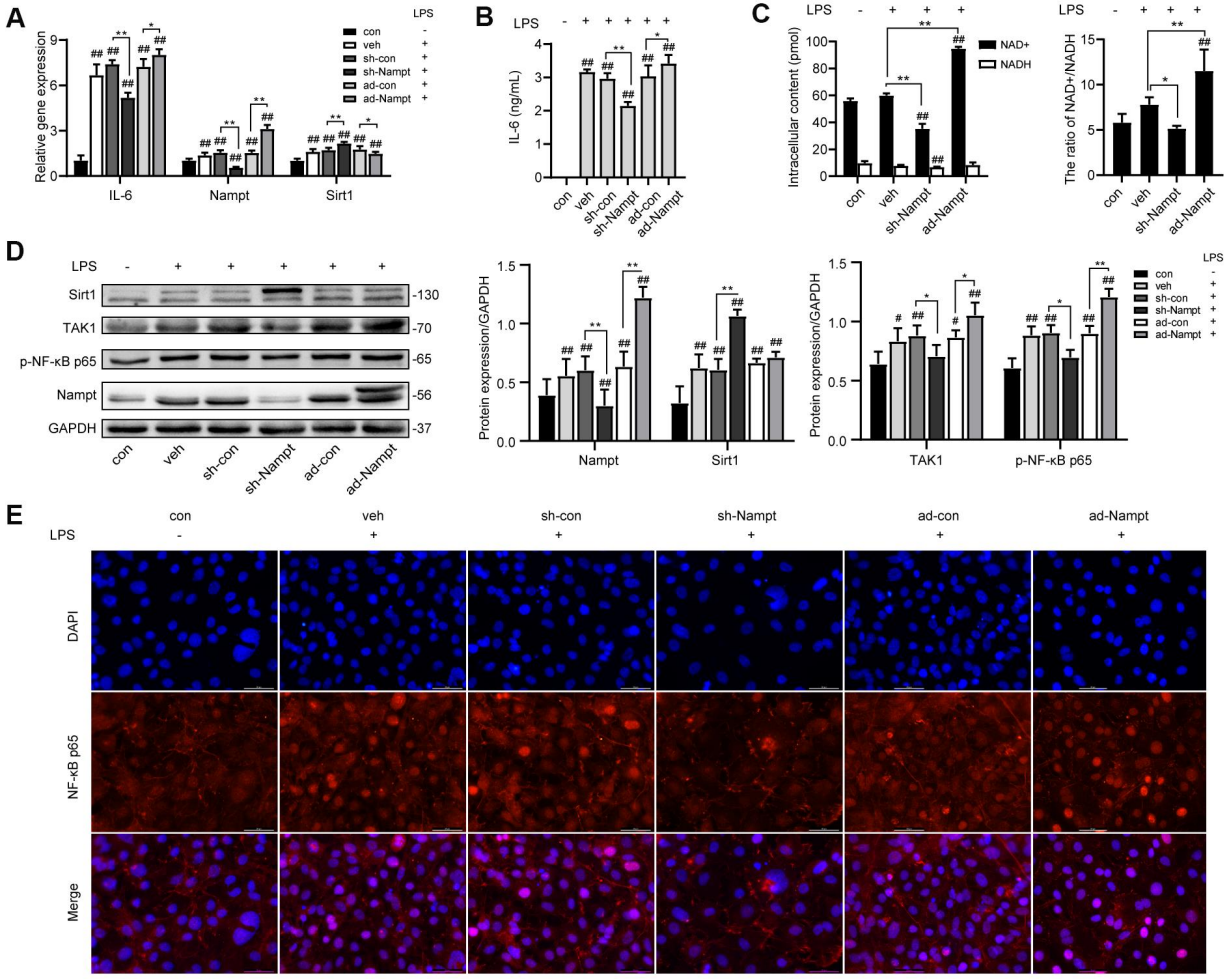
**Nampt promoted LPS-induced inflammation in MC3T3-E1 cells.** After treatment for 3 days, (**A**) Real-time PCR analysis showing the mRNA expression of IL-6, Nampt and Sirt1 normalized to β-actin. (**B**) ELISA result showing the IL-6 levels. (**C**) NAD^+^/NADH analysis showing intracellular content of NAD^+^ and NADH, and their ratio. (**D**) Western blot analysis showing the protein expression of Nampt, Sirt1, TAK1 and p-NF-κB p65. (**E**) The nuclear translocation of NF-κB p65 was measured by fluorescence immunocytochemistry (200×). Red, NF-κB p65; blue, DAPI. The data are expressed as the mean ± SD. *, *p*<0.05; **, *p*<0.01. #, *p*<0.05 vs. con; ##, *p*<0.01 vs. con.

To investigate the relationship between Nampt and IL-6 secretion, control cells and cells transfected with adenoviruses were cultured without or with 100 ng/mL LPS in OBM for 3 days. As shown in Figure 3A, the mRNA level of IL-6 and Nampt was significantly increased after LPS treatment, and further increased after Nampt overexpression, but decreased in the Nampt knockdown group. The IL-6 concentration in culture supernatants (Figure 3B) and protein level of Nampt (Figure 3D) were consistent with the change. Sirt1 expression was significantly elevated after LPS treatment and further increased following Nampt knockdown, but decreased after Nampt overexpression (Figures 3A, 3D). The intracellular NAD^+^ content was decreased after Nampt knockdown and increased after Nampt overexpression. The NADH content was decreased after Nampt knockdown. LPS treatment significantly elevated the NAD^+^/NADH ratio. Nampt overexpression cells had significant higher NAD^+^/NADH ratio than that of con and veh cells, while inhibiting Nampt decreased the NAD^+^/NADH ratio (Figure 3C).

### Nampt increased nuclear factor-κappaB (NF-κB) activation

Since NF-κB signal pathway was an important regulator of inflammation, we used western blot analysis and fluorescence immunocytochemistry to examine the effect of LPS on activation of NF-κB signaling in MC3T3-E1 cells. 100 ng/mL LPS increased the protein level of TGF-β-activated kinase 1 (TAK1) and p-NF-κB p65 in LPS-treated cells compared to non-treated cells. Nampt overexpression significantly increased TAK1 and p-NF-κB p65 expression in LPS-treated cells, while Nampt knockdown significantly reversed the increase of TAK1 and p-NF-κB p65 expression (Figure 3D). In addition, Nampt overexpression enhanced the nuclear translocation of NF-κB p65, while knockdown of Nampt inhibited the nuclear translocation of NF-κB p65 after LPS treatment (Figure 3E). These results indicated that Nampt promoted LPS-induced inflammation in MC3T3-E1 cells.

### Overexpression of Nampt rescued LPS-induced inhibition on osteogenic differentiation

**Figure 4:**
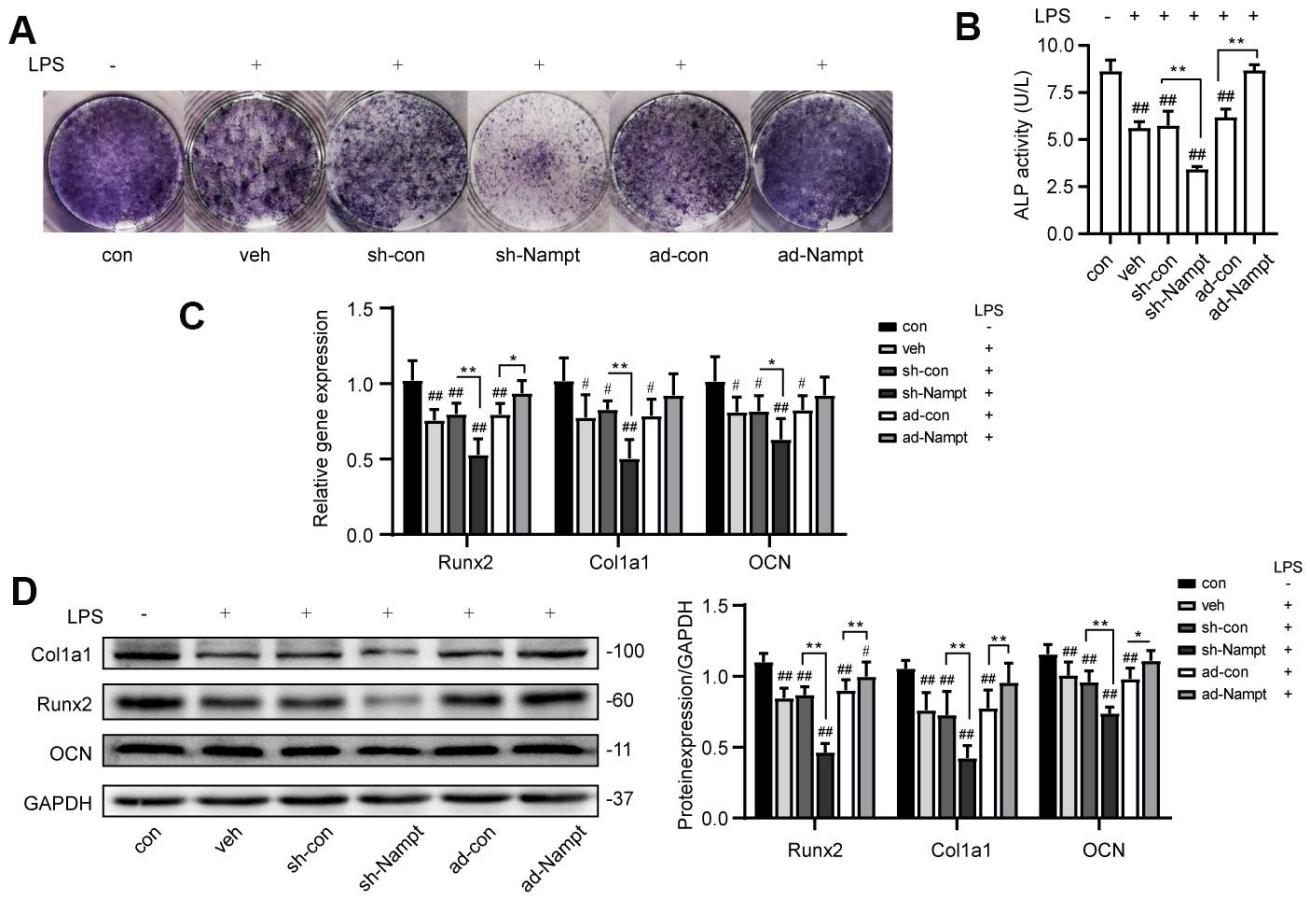
**Overexpression of Nampt rescued LPS-induced inhibition on osteogenic differentiation.** On the 7^th^ day, (**A**) ALP staining and (**B**) Measurement of ALP activity showing that ALP was increased in ad-Nampt cells. (**C**) Real-time PCR analysis showing Runx2, Col1a1 and OCN gene expression normalized to β-actin. (**D**) Western blot results showing the protein expression of Runx2, Col1a1 and OCN. The data are expressed as the mean ± SD. *, *p*<0.05; **, *p*<0.01. #, *p*<0.05 vs. con; ##, *p*<0.01 vs. con.

To explore the effect of Nampt in osteogenesis in the presence of inflammation, control cells, sh-Nampt cells and ad-Nampt cells were cultured with 100 ng/mL LPS in OBM for 7 days. LPS decreased the activity of ALP, the expression of ALP, Runx2, Col1a1 and OCN. PCR and western blot showed that Nampt knockdown further decreased the expression of these osteogenic markers, however, overexpression of Nampt significantly increased the expression of ALP, Runx2, Col1a1 and OCN. ALP activity, PCR and western blot analysis showed that there was no significant difference between con cells cultured in OBM and ad-Nampt cells treated with LPS (Figure 4). Overexpression of Nampt overrode the effect of LPS and rescued LPS-induced inhibition on osteogenic differentiation.

### FK866 inhibited NF-κB activation and osteogenic differentiation

**Figure 5:**
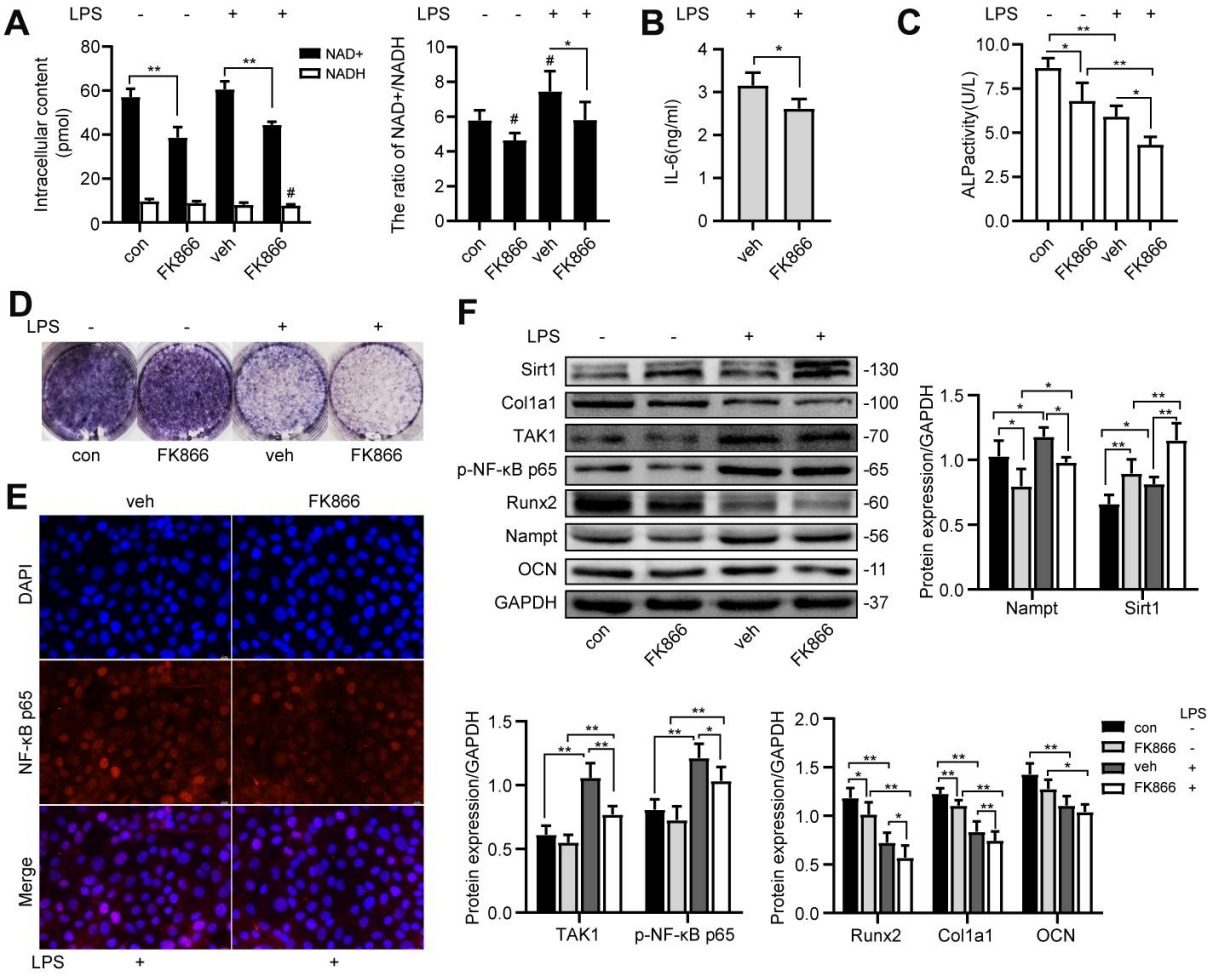
**FK866, a Nampt inhibitor, inhibited IL-6 induced inflammation and osteogenic differentiation.** After treatment with 1 nM FK866 and 100 ng/mL LPS for 3 days, (**A**) NAD^+^/NADH analysis showing intracellular content of NAD^+^ and NADH, and their ratio. (**B**) ELISA result showing the IL-6 levels. (**E**) The nuclear translocation of NF-κB p65 was measured by fluorescence immunocytochemistry (200×). Red, NF-κB p65; blue, DAPI. On the 7^th^ d of culture, (**C**) Measurement of ALP activity and (**D**) ALP staining showing that ALP was decreased after treatment with FK866. (**F**) Western blot results showing the protein expression of Nampt, Sirt1, TAK1, p-NF-κB p65, Runx2, Col1a1 and OCN. The data are expressed as the mean ± SD. *, *p*<0.05; **, *p*<0.01. #, *p*<0.05 vs. con.

FK866, a Nampt inhibitor, decreased the intracellular NAD^+^ content, the NAD^+^/NADH ratio (Figure 5A) and the protein level of Nampt (Figure 5F) with or without LPS treatment. The IL-6 secretion (Figure 5B), the nuclear translocation of NF-κB p65 (Figure 5E) and the protein level of TAK1 and p-NF-κB p65 (Figure 5F) were inhibited after FK866 treatment. FK866 elevated Sirt1 expression with or without LPS treatment (Figure 5F). Moreover, FK866 decreased the expression of osteogenic markers (Figure 5C, 5D, 5F). These results showed that inhibition of Nampt by 1 nM FK866 had the same inhibitory effect on NF-κB activation and osteogenic differentiation as Nampt knockdown.

### Inhibiting Sirt1 decreased LPS-induced NF-κB activation and osteogenic differentiation

To explore the effect of Sirt1 on LPS-induced NF-κB activation and osteogenic differentiation in MC3T3-E1 cells, EX527 was used to inhibit Sirt1 expression in LPS-treated cells. Cells were cultured in medium for 3 days after adenovirus transfection, then 100 ng/mL LPS was applied with or without 30 μM EX527 in OBM for indicated days.

**Figure 6:**
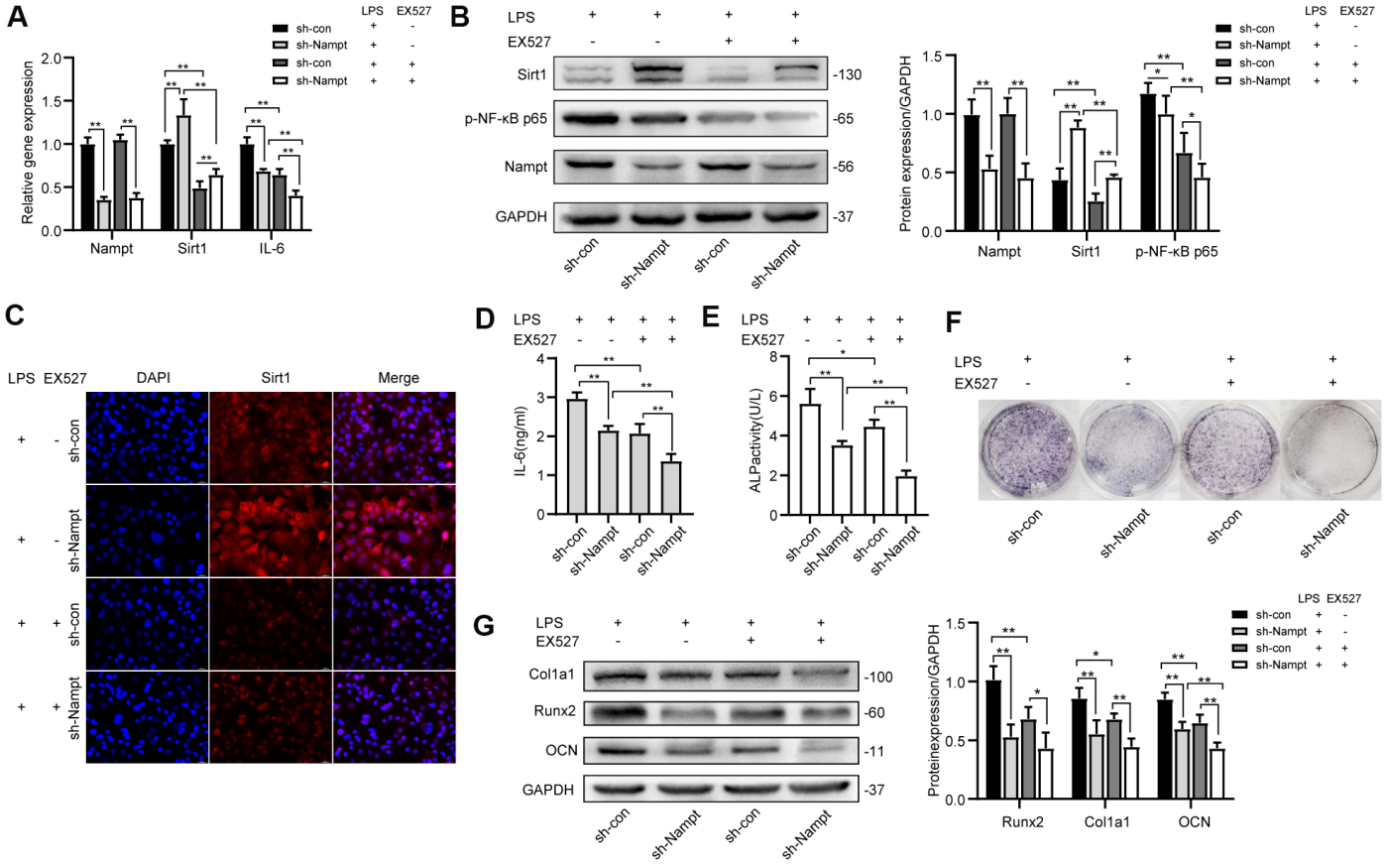
**EX527, a selective Sirt1 inhibitor, decreased LPS-induced IL-6 secretion and osteogenic differentiation after LPS treatment.** After treatment for 3 days, (**A**) Real-time PCR analysis showing the mRNA expression of Nampt, Sirt1, and IL-6 normalized to β-actin. (**B**) Western blot analysis showing the protein expression of Nampt, Sirt1, and p-NF-κB p65. (**C**) The expression of Sirt1 was measured by fluorescence immunocytochemistry (200×). Red, Sirt1; blue, DAPI. (**D**) ELISA results showing the IL-6 levels. After EX527 was applied for 7 days, (**E**) ALP staining and (**F**) Measurement of ALP activity were decreased. (**G**) Western blot analysis showing the protein expression of Runx2, Col1a1 and OCN. The data are expressed as the mean ± SD. *, *p*<0.05; **, *p*<0.01.

After 3 days, Real-time PCR (Figure 6A), western blotting (Figure 6B) and fluorescence immunocytochemistry (Figure 6C) showed that Sirt1 expression was significantly attenuated after EX527 treatment, and we observed no change in Nampt expression at mRNA and protein level (Figures 6A, 6B). Real-time PCR and ELISA results (Figures 6A, 6D) showed that IL-6 secretion induced by LPS was significantly decreased after EX527 was applied. Protein level of p-NF-κB p65 was decreased after EX527 treatment (Figure 6B). These results indicated that NF-κB activation was attenuated after Sirt1 inhibition.

After 7 days, the expression and activity of ALP were attenuated after EX527 treatment (Figures 6E, 6F). Western blot results showed that the expression of OCN was significantly decreased after EX527 was applied, both in sh-con cells and in Nampt knockdown cells. The expression of Runx2 and Col1a1 was inhibited in sh-con cells after EX527 was applied (Figure 6G).

### NMN and SRT 1720 rescued LPS-induced inhibition on osteogenic differentiation

**Figure 7:**
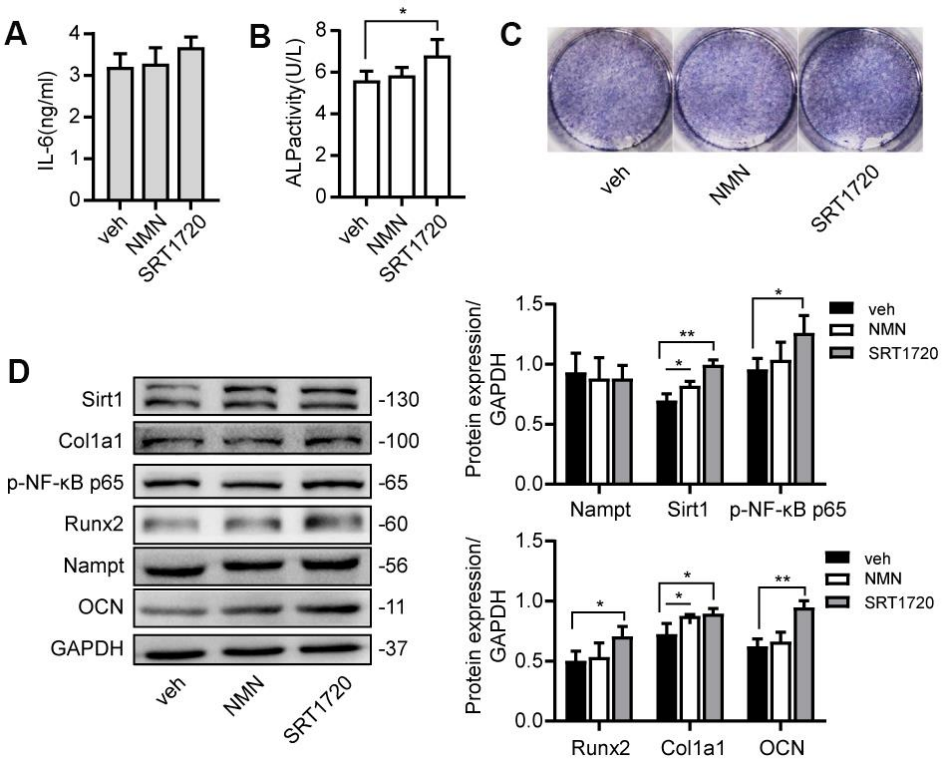
**NMN and SRT 1720 promoted osteogenic differentiation after LPS treatment. Cells were cultured in 100 μM NMN or 5 μM SRT 1720 with 100 ng/mL LPS for 7 days.** (**A**) ELISA results showing the IL-6 levels. (**B**) ALP activity and (**C**) ALP staining were increased after incubation with SRT 1720. (**D**) Western blot results showing the protein expression of Nampt, Sirt1, p-NF-κB p65, Runx2, Col1a1 and OCN. The data are expressed as the mean ± SD. *, *p*<0.05; **, *p*<0.01.

Cells were cultured with NAD^+^ precursor NMN or Sirt1 agonist SRT 1720 for 7 days. There was no significant change of Nampt expression (Figure 7D). NMN upregulated the protein level of Sirt1 and Col1a1. SRT 1720 increased ALP activity, expression of p-NF-κB p65, Runx2, Col1a1 and OCN (Figure 7B, 7D). These results showed that NMN and SRT 1720 rescued LPS-induced inhibition on osteogenic differentiation.

Taken together, these results suggested that Nampt and Sirt1 exerted additive effect on inflammation and osteogenic differentiation after LPS treatment in MC3T3-E1 cells. Overexpression of Nampt or Sirt1 overrode the effect of LPS and rescued LPS-induced inhibition on osteogenic differentiation.

## DISCUSSION

Published evidence suggests that Nampt affects the osteoblastic lineage fate and is involved in innate immunity. Chronic inflammation perturbs bone metabolism and promotes bone loss [16]. In this study, we demonstrated that Nampt acted both in promoting osteoblast differentiation and in advancing inflammatory response in preosteoblasts

LPS is a major immunomodulatory and structural component in the outer membrane of gram-negative bacteria [17]. Excessive activation of toll-like receptor 4 (TLR4) by LPS stimulates the downstream NF-κB signaling pathways and triggers the subsequent inflammatory cytokine storm [18]. In our study, LPS was used to construct an inflammatory cell model. IL-6 has a wide range of biological activities in immune regulation and inflammation [19]. Estrogen deficiency following menopause causes an age-related increase in IL-6, even in the absence of infection, trauma or stress [20, 21]. IL-6 gene knockout mice are protected from cancellous bone loss induced by estrogen depletion [22]. These evidences indicate that IL-6 may play an important role in bone metabolism. In our study, LPS activated NF-κB signaling, induced IL-6 production by autocrine stimulation of MC3T3-E1 cells, decreased cell proliferation and diminished ALP activity *in vitro*. NF-κB activation suppresses osteoblast activities [16, 23]. The osteoblast-producing IL-6 may further inhibit the activities and differentiation of osteoblasts. The inhibition of IL-6 on osteoblasts may occur through the suppression of mitogen-activated protein kinase (MAPK) activities by activating signal transducers and activators of transcription (STATs), through the effects of SMAD ubiquitylation regulatory factor 1 (SMURF1) and SMURF2 and the upregulation of Dickkopf-related protein 1 (DKK1) and sclerostin (SOST) which inhibit the Wnt-frizzled signaling pathway, resulting in downregulation of many other osteogenic gene products [8].

The osteoblast lineage cells include the group of mesenchymal progenitors, preosteoblasts, osteoblasts (mature osteoblasts), bone-lining cells and osteocytes [24]. The rate of bone formation is controlled by the speed and capacity of precursor cells differentiating into mature osteoblasts and by their life span [25]. Proinflammatory cytokines are mediators of inflammation-associated osteoporosis [8]. The degree of the inflammatory response is associated with the extent of local and systemic bone loss [3, 8].

Nampt is a multifaceted molecule, and its functions depend on cellular and genetic context [26]. Nampt promotes osteogenesis and decreases adipogenesis in mesenchymal stem cells [27, 28]. In our study, Nampt knockdown inhibited the expression of osteogenic markers ALP, Runx2, Col1a1 and OCN in preosteoblasts. In recent years, Nampt was found as a novel biomarker in multiple inflammatory diseases. Seo et al. found that plasma Nampt concentrations were positively associated with circulating IL-6 levels in nondiabetic healthy Korean women [29]. Pulla et al. reported that Nampt inhibitors were helpful in treating inflammation by reducing the expression of various cytokines, such as IL-6 and TNF-α [30]. However, the role of Nampt in inflammatory bone loss has not been clearly elucidated. Our study showed that when cells were treated with varying concentrations of LPS, the upregulation of Nampt was consistent with the increase of IL-6 secretion. It indicated that Nampt level was associated with the degree of inflammatory response in MC3T3-E1 cells. Because high concentrations of LPS inhibited cell proliferation and differentiation, and 100 ng/mL induced the highest expression level of IL-6 and Nampt among all groups, we selected 100 ng/mL as the appropriate concentration for subsequent experiments.

We found that Nampt knockdown reversed LPS-induced increase of IL-6 secretion. Expression of molecules in NF-κB signaling, TAK1 and p-NF-κB p65, were also decreased. Nampt knockdown inhibited the nuclear translocation of NF-κB p65. While overexpression of Nampt activated NF-κB signaling. These findings demonstrated that Nampt promoted the LPS-induced inflammatory response of MC3T3-E1 cells.

After LPS treatment, overexpression of Nampt enhanced the osteoblast differentiation, suggesting that Nampt sustained its effect in enhancing osteogenesis in the presence of inflammation. Overexpression of Nampt overrode the effect of LPS and rescued LPS-induced inhibition on osteogenic differentiation. Thus, Nampt was a potential therapeutic target in inflammation-associated bone loss.

Castro et al found that, by directly targeting Nampt mRNA expression, NAD levels and Sirt1 activity are reduced in the liver [31]. Friebe et al. found that increased Nampt levels mediated by Sirt1 induction delays apoptosis of granulocytes and thus maintains inflammation [12]. In our study, when we suppressed Sirt1 expression by using the selective inhibitor EX527, the extent of the inflammatory response was attenuated, resulting in decreased expression of p-NF-κB p65 and IL-6 secretion. Sirt1 also affected the osteogenesis of MC3T3-E1 cells. EX527 inhibited the expression of ALP, Runx2, Col1a1 and OCN, with or without Nampt inhibition. Nampt and Sirt1 seemed to exert additive effect in the inflammatory response and osteogenic differentiation in preosteoblasts.

Crosstalk among inflammatory cells and cells related to bone healing is essential in bone formation [7]. The activity of bone-forming osteoblasts is coupled to that of bone-resorbing osteoclasts [32]. IL-6 leads to massive upregulation of osteoclast differentiation and activation by the JAK-STAT pathway with or without the presence of receptor activator of NF-κB ligand (RANKL) [8]. LPS induced the secretion of IL-6 in preosteoblasts, and therefore osteoclast differentiation and activation could be amplified [33]. Nampt may be a potential molecule linking the differentiation and activation of osteoblasts and osteoclasts in chronic inflammation via IL-6 secretion.

In summary, LPS could induce IL-6 secretion via activation of NF-κB signaling pathway, which was mediated by Nampt and Sirt1 upregulation in MC3T3-E1 cells. Nampt and Sirt1 also exerted additive effect in osteogenic differentiation. Overexpression of Nampt or Sirt1 overrode the effect of LPS and rescued LPS-induced inhibition on osteogenic differentiation. It indicates that Nampt may be a potential therapeutic target to modulate inflammatory response in inflammation-associated bone loss.

## MATERIALS AND METHODS

### Cell culture

MC3T3-E1, a murine osteoblastic cell line, was purchased from the Shanghai Cell Bank of the Chinese Academy of Sciences (MC3T3-E1 Subclone 14). Cells were cultured in MEM alpha modification medium (α-MEM) (HyClone) supplemented with 10% fetal bovine serum (FBS) (Excell) and 1% penicillin and streptomycin (Beyotime) in a humidified incubator with 5% CO_2_ at 37° C. For osteoblast induction, cells were cultured in OBM after cells reached 90% confluence. OBM was prepared with α-MEM medium supplemented with 10% FBS, 10 mM β-glycerophosphate, 50 μg/mL α-ascorbic acid, and 0.1 μM dexamethasone, and the medium was changed every 3 days. For the induction of inflammation, lyophilized LPS powder (Sigma, L2630, Escherichia coli O111:B4) was diluted with cell culture medium to 1 mg/mL for storage and further diluted to the working concentration. FK866 (Beyotime, SD7257) was diluted with cell culture medium to 1 nM. EX527 (Beyotime, SC0281), a selective Sirt1 inhibitor, was diluted with cell culture medium to 30 μM. NMN (Sigma, N3501), the NAD^+^ precursor, was diluted with cell culture medium to 100 μM. SRT 1720 (Beyotime, SC0267), a selective Sirt1 agonist, was diluted with cell culture medium to 5 μM.

### CCK-8 assay

Cell proliferation was measured using a CCK-8 cytotoxicity assay (Dojindo, CK04). 2000 cells were seeded per well in a 96-well plate and incubated with 5% CO_2_ at 37° C for 24 h. Then the cells were treated with 20, 50, 100, 200, 500 and 1000 ng/mL LPS or without LPS for 3 days. Before measurement, 10 μL CCK-8 reagent was added per well and incubated for 40 min at 37° C in a humidified incubator, then the absorbance was measured at 450 nm using a microplate reader (Thermo Fisher Scientific). Each group was tested in quintuplicate.

### Measurement of NAD^+^ and NADH content

Cells were seeded in a 6-well plate. Intracellular content of NAD^+^ and NADH and their ratio were measured with a NAD^+^/NADH assay kit (Beyotime, S0175). NAD^+^/NADH were extracted from cell samples with 400 μL of extraction buffer on ice and then supernatant was collected. For each sample, 100 μL of the extract was heated to 60° C for 30 minutes in a water bath. All NAD^+^ was decomposed while the NADH was still intact. Both heated and unheated extracts from each sample, together with the NADH standard solutions, were transferred into a 96-well plate at 20 μL/well in triplicate. Then 90 μL of alcohol dehydrogenase working solution was added to each well and incubated at 37° C for 10 min to convert NAD^+^ to NADH. Then 10 μL/well of stop solution was added into each well. The absorbance was measured at 450 nm using a microplate reader (Thermo Fisher Scientific). The amount of NAD^+^ from each sample was calculated as [NAD_total_ (values from the unheated extracts)] minus [NADH (values from the heated extracts)] and then divided by the protein concentration. [NAD^+^]/[NADH]=([NAD_total_]-[NADH])/[NADH].

### ALP staining

Cells were seeded in a 24-well plate at a density of 3000/well to induce osteogenic differentiation for 7 days. The cells were fixed with 4% paraformaldehyde at room temperature for 5 min. Then a BCIP/NBT ALP color development kit (Beyotime, C3206) was used to stain ALP for 30 min at room temperature in dark.

### Measurement of ALP activity

Cells were seeded in a 24-well plate at a density of 3000/well to induce osteogenic differentiation for 7 days. Proteins were extracted with 300 μL/well cell lysis buffer without inhibitors (Beyotime, P0013J) and then centrifuged at 12000 r/m for 3 min. Collect supernatants and analyze the ALP activity by an ALP assay kit (Beyotime, P0321) using p-nitrophenyl phosphate (*p*NPP) as a phosphatase substrate. After the mixture was incubated for 10 min at 37° C, the absorbance of each well was measured at 405 nm on the microplate reader (Thermo Fisher Scientific). The relative ALP activity was referred to the amount of enzyme causing the hydrolysis of one micromole of *p*NPP per minute at pH 9.8 and 37° C (diethanolamine buffer).

### Adenovirus and transfection of MC3T3-E1 cells

The adenovirus that was used to knock down and over express mouse Nampt was purchased from Hanbio Biotechnology. Cells were seeded at a density of 1×10^5^/mL and cultured overnight. The confluence reached 50%~60% by the next day, and the cells were transfected with sh-Nampt, ad-Nampt or empty virus (negative control, sh-con and ad-con) at a multiplicity of infection (MOI) of 300 and incubated at 37° C for 6 h. Then the cells were cultured with fresh medium for indicated days. Nampt expression was examined at both the mRNA and protein levels.

### ELISA

MC3T3-E1 cells were seeded in a 12-well plate. To explore the effect of LPS on secretion of proinflammatory cytokines, cells were cultured with 20, 50, 100, 200, 500 and 1000 ng/mL LPS or without LPS for 3 days. To explore the relationship between Nampt and inflammation, cells transfected with sh-con (300 MOI), sh-Nampt (300 MOI), ad-con (300 MOI) or ad-Nampt (300 MOI) were cultured with 100 ng/mL LPS for 3 days. The amount of IL-1β, IL-6 and TNF-α was determined in culture supernatants with the ELISA kits (4A Biotech: IL-6, CME0006; IL-1β, CME0015; and TNF-α, CME0004). Briefly, culture supernatants were purified and then diluted 10-fold, and the dilutions were added by 100 μL/well to an ELISA plate. Each group was tested in triplicate. The absorbance of the wells was measured at 450 nm on the microplate reader (Thermo Fisher Scientific). The IL-6, IL-1β and TNF-α concentrations were estimated by reference to standard curves, which were generated using standard proteins. The experiment was repeated for three times.

### Real-time PCR

Extraction of total RNA was performed with TRIzol reagent (Tiangen). The RNA was reverse transcribed using the PrimeScript RT reagent kit with gDNA Eraser (TAKARA, RR047A), and the resulting cDNA was amplified by PCR in an ABI-7500 PCR system (Thermo Fisher Scientific) using TB Green Premix Ex Taq II (TAKARA, RR820A). Transcript levels of all target genes were normalized to those of β-actin and are expressed as fold changes relative to the indicated controls, according to the comparative 2^-ΔΔCt^ value method, where *ΔΔ=ΔC*t_target_*-ΔCt*_β-actin_. The primer sequences (TAKARA) used are listed in Table 1.

**Table 1:** Primer sequences of mRNA.

**Genes**	**Forward**	**Reverse**
β-actin	5’-AGATTACTGCTCTGGCTCCTAGC-3’	5’- ACTCATCGTACTCCTGCTTGCT-3’
Nampt	5’-GTTCTGGTGGCGCTTTGCTA-3’	5’-AGTTCCCCGCTGGTGTCCTA-3’
Sirt1	5’-AGGGAACCTTTGCCTCATCTAC-3’	5’-GGTGGCAACTCTGATAAATGAAC-3’
IL-6	5’-CTTGGGACTGATGCTGGTGAC-3’	5’-TTCTCATTTCCACGATTTCCCA-3’
TNF-α	5’-GCACCACCATCAAGGACTCAA-3’	5’-CAGGGAAGAATCTGGAAAGGTC-3’
IL-1β	5’-GTGAAATGCCACCTTTTGACAGT-3’	5’-AATGAGTGATACTGCCTGCCTGA-3’
Runx2	5’-CCAACTTCCTGTGCTCCGTG-3’	5’-ATAACAGCGGAGGCATTTCG-3’
Col1a1	5’-TGGCGGTTATGACTTCAGCTT-3’	5’-CTCAAGGTCACGGTCACGAAC-3’
OCN	5’-TGAACAGACTCCGGCGCTAC-3’	5’-AGGCGGTCTTCAAGCCATACT-3’

### Western blot analysis

Extraction of total proteins was performed with RIPA lysis buffer (Beyotime, P0013B) supplemented with 1% PMSF (Beyotime, ST506) on ice. Protein concentrations were measured with an enhanced BCA protein assay kit (Beyotime, P0010S). 40 μg proteins per sample were separated via electrophoresis and were transferred onto polyvinylidene difluoride membranes (PVDF, Life Technologies). The membranes were blocked in TBST containing 5% nonfat milk or 5% BSA (Beyotime) for 1 h, followed by incubation at 4° C overnight with primary antibodies, and then horseradish peroxidase (HRP)-conjugated anti-rabbit IgG secondary antibody at 37° C for 1 h. The following antibodies were used: rabbit polyclonal anti-Nampt (Abcam, ab45890), rabbit monoclonal anti-Sirt1 (Abcam, ab189494), rabbit polyclonal anti-Runx2 (Affinity, AF5186), rabbit polyclonal anti-Col1α1 (Affinity, AF7001), rabbit polyclonal anti-OCN (Affinity, DF12303), rabbit polyclonal anti-TAK1(Affinity, AF7616), rabbit polyclonal anti-NF-κB p65 (Affinity, AF5006), rabbit polyclonal anti-phospho-NF-κB p65 (Affinity, AF2006), rabbit polyclonal anti-GAPDH (Goodhere, AB-P-R001), and HRP-labeled goat anti-rabbit IgG(H+L) (Beyotime, A0208). Protein bands were detected using a hypersignal ECL kit (4A Biotech, 4AW012-100) on a chemiluminescence detection system (Vilber). Band density was analyzed with ImageJ software (National Institutes of Health, Bethesda, MD, USA).

### Fluorescence immunocytochemistry

The cells were fixed with 4% paraformaldehyde at room temperature for 10 min, permeabilized in 0.3% Triton X-100 (Solarbio) for 10 min and then blocked with 10% normal goat serum (Boster, AR0009) for 30 min at 37° C. The cells were then incubated overnight at 4° C with rabbit anti-Sirt1 (Abcam, ab189494) at a 1/100 dilution or rabbit anti- NF-κB p65 (Affinity, AF5006) at a 1/200 dilution. A Cy3-conjugated goat anti-rabbit IgG (H+L) (Earthox, E031620-01, Red) secondary antibody at a 1/200 dilution was used. Nuclear DNA was labeled with DAPI (blue). Images were taken with a fluorescence microscope (Leica, DM6000B).

### Statistical analysis

All data are expressed as the mean ± SD. Differences between groups were compared with one-way ANOVA with a post hoc *t* test. A *p* value of <0.05 was considered statistically significant. GraphPad Prism 8.0.1 software (GraphPad Software Inc., San Diego, CA) and Statistical software package IBM SPSS Statistics 23.0 (IBM Corp., Armonk, New York, USA) were used for data analysis.
